# Effective treatment with rituximab for the maintenance of remission in frequently relapsing minimal change disease

**DOI:** 10.1111/nep.12744

**Published:** 2016-09-12

**Authors:** Eugenia Papakrivopoulou, Ali M. Shendi, Alan D. Salama, Maryam Khosravi, John O. Connolly, Richard Trompeter

**Affiliations:** ^1^ University College London Centre for Nephrology, Royal Free CampusUniversity College LondonLondonUK; ^2^ University College London Institute of Child HealthUniversity College LondonLondonUK; ^3^ Nephrology Unit, Internal Medicine Department, Faculty of MedicineZagazig UniversityZagazigEgypt

**Keywords:** calcineurin inhibitor, glucocorticoid, minimal change disease, nephrotic syndrome, rituximab

## Abstract

**Aim:**

Treatment of frequently relapsing or steroid‐dependent minimal change disease (MCD) in children and adults remains challenging. Glucocorticoids and/or other immunosuppressive agents are the mainstay of treatment, but patients often experience toxicity from prolonged exposure and may either become treatment dependent and/or resistant. Increasing evidence suggests that rituximab (RTX) can be a useful alternative to standard immunosuppression and allow withdrawal of maintenance immunosuppressants; however, data on optimal treatment regimens, long‐term efficacy and safety are still limited.

**Methods:**

We undertook a prospective study of RTX to allow immunosuppression minimization in 15 young adults with frequently relapsing or steroid‐dependent, biopsy‐proven MCD. All patients were in remission at the start of treatment and on a calcineurin inhibitor. Two doses of RTX (1 gr) were given 6 months apart. A subset of patients also received an additional dose 12 months later, in order to examine the benefit of re‐treatment. Biochemical and clinical parameters were monitored over an extended follow‐up period of up to 43 months.

**Results:**

Median steroid‐free survival after RTX was 25 months (range 4–34). Mean relapse frequency decreased from 2.60 ± 0.28 to 0.4 ± 0.19 (*P* < 0.001) after RTX. Seven relapses occurred, five of which (71%) when CD19 counts were greater than 100 µ. Immunoglobulin levels remained unchanged, and no major side effects were observed throughout the follow‐up period.

**Conclusions:**

Rituximab therapy is effective at maintaining prolonged steroid‐free remission and reducing relapse frequency in this group of patients. Our study lends further support for the role of RTX in the treatment of patients with frequently relapsing or steroid‐dependent MCD.

## Summary at a Glance

Treatment of steroid‐dependent frequent relapsing nephrotic syndrome is challenging issue. The manuscript examining the efficacy and safety of treatment of rituximab for those patinets give us meaning information in the field of clinical nephrology.

Minimal change disease (MCD) is the most common cause of nephrotic syndrome (NS) in children and accounts for up to 20% of NS in adults 1. The disease derives its name from the normal (minimal change) appearance of glomeruli on light and immunofluorescence microscopy due to lack of immunoglobulin or complement deposition. Electron microscopy, however, reveals the loss of the highly branched podocyte morphology with widespread effacement of foot processes. Although the exact aetiology of MCD remains unknown, dysregulation of the immune system is thought to be an important factor in its pathogenesis 2.

Steroids are the mainstay of treatment for MCD, and approximately, 80% of patients will respond to an initial course of prednisolone. However, relapse rates are high, especially in younger patients (<40 years old) 3, and between 56% and 76% of patients will experience at least one relapse with tapering or withdrawal of the initial steroid therapy. Relapses are also seen in 40% of adults who had MCD during childhood 3, 4, 5. Repeated courses of steroids are needed for recurrent relapses with significant resultant morbidity and mortality 6, 7. Frequent relapses and hospital visits also impact adversely on work, education and overall quality of life 8, 9. Immunosuppressive (IS) agents, such as calcineurin inhibitors (CNIs) (cyclosporine A /tacrolimus), cyclophosphamide and mycophenolate mofetil, are often used in an attempt to reduce relapse frequency and therefore avoid the severe adverse side effects of steroid treatment. However, prolonged use of CNI can result in side effects including nephrotoxicity, hirsuitism, hypertension and hyperlipidaemia 10, 11. Moreover, at least 30% of patients continue to suffer frequent relapses despite repeated courses of the aforementioned therapies and can remain steroid dependent with relapses of NS following discontinuation of steroids, or during the tapering phase of treatment 3. These patients have increased risk of complications because of the use of repeated courses of steroids and other IS agents (in many cases for >15–20 years) and are therefore in urgent need of a more effective and safe treatment.

Rituximab (RTX) is a chimeric monoclonal antibody inhibiting CD‐20 mediated B‐cell proliferation and differentiation, successfully used in non‐Hodgkin lymphoma, systemic diseases such as rheumatoid arthritis or severe anti‐neutrophil cytoplasmic antibody‐associated vasculitis and renal disorders such as membranous nephropathy 12, 13. In children, RTX has been used since 2006 to treat frequently relapsing NS and, in 2012, was included in the Kidney Disease: Improving Global Outcomes *Guidelines for the Treatment of Glomerulonephritis in Children,* as an option for the treatment of this challenging group of patients 14. In adults, increasing evidence suggests that RTX is capable of reducing relapse frequency and concomitant immunosuppression in 65–85% of patients 15, 16, 17, 18, 19. However, its use has been very varied: different dosage regimes, in mixed populations (steroid dependent and/or steroid resistant) and for differing indications (induction or maintenance of remission). As a result, it is still not clear whether RTX is best used to induce remission or to maintain it, what the optimal dose should be and whether repeated doses improve response rates and prolong remission. Additional data are therefore needed to inform clinical practice on how best to use RTX in this patient population, so that definitive randomized trials can be planned. The aim of this study was to evaluate the efficacy and safety of a standard dose of RTX in maintaining remission, reducing relapse frequency and withdrawing immunosuppression, in a group of adult patients with frequently relapsing and steroid‐dependent MCD.

## Methods

### Patient population and follow up

Fifteen adult patients (18–46 years old), nine male and six female, fulfilling the following criteria were enrolled in this study:

1. Biopsy‐proven MCD
2. Frequently relapsing or steroid‐dependent disease. Frequent relapse was defined as two or more relapses within 1 year. Steroid dependence was defined as relapse upon tapering steroid therapy or within 4 weeks of discontinuing steroids and need for long‐term maintenance steroids
3. Maintenance immunosuppression with a CNI (cyclosporine A or tacrolimus)
4. Remission of NS defined as proteinuria <3.5 g/day
5. No previous RTX treatment

We enrolled patients consecutively from December 2011 until July 2014 when funding for RTX, for this indication, was restricted in the UK. Clinical and laboratory data were obtained at baseline, 6, 12, 18 months and every 6 months after that until 36 months. Laboratory data included serum albumin levels, urine protein/creatinine ratio, immunoglobulin levels and CD19 cell counts. Follow‐up period for the group as a whole was 12–43 months (median 20) and concluded in July 2015. The study conformed to the ethical standards of the Declaration of Helsinki, and all subjects gave informed consent prior to inclusion in the study.

### Treatment

**Figure 1:**
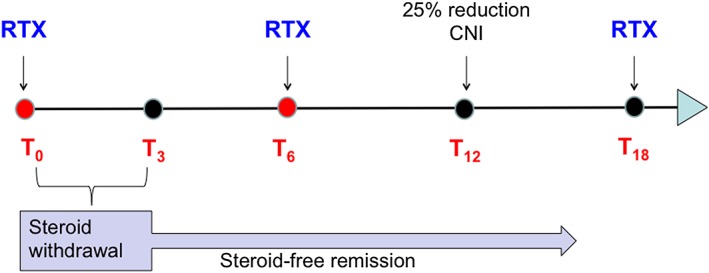
Schematic representation of the treatment protocol. All patients at T0 were given 1 gr rituximab (RTX). Steroids were tapered and withdrawn within 3 months (T3) if applicable. A second RTX dose was given 6 months later (T6). At 1 year (T12), calcineurin inhibitor (CNI) was reduced by 25% if no relapses had occurred. Four patients received an additional RTX dose 18 months after the first dose (T18).

The protocol for RTX administration is shown in Figure 1. A standard dose of 1 gr was used for all patients. Twelve patients received two doses at an interval of 6 months (T0 and T6). Three patients received only a single dose because of funding restrictions. Four patients received an additional RTX dose, at 18 months in order to evaluate the safety and efficacy of re‐treatment (T18). Where appropriate, data for these groups are presented separately.

In order to minimize infusion reactions, 40 mg methylprednisolone, 10 mg Piriton and 1 gr Paracetamol were administered prior each RTX infusion. B‐cell depletion, defined as a peripheral blood CD19 cell count of <5 cells/µl, was confirmed 2 weeks post‐treatment and at every follow‐up visit (normal range in our laboratory defined as 100–500 cells/µl). Patients who did not deplete their B cells were re‐treated 4 weeks later. If patients were on concomitant steroids, they were tapered and withdrawn by 3 months following the first RTX dose. At 12 months, if no relapse had occurred, CNI dose was reduced by 25%. Following the initial reduction, CNI dose was reduced by 25% at six monthly intervals. NS relapse was treated as per standard protocol in our unit with 1 mg/kg prednisolone (max 60 mg/day) until remission, followed by gradual weaning over 8–12 weeks and reinstatement of the original CNI dose. No change in other medications (ACE inhibitors or ARBs) was introduced throughout the study period.

### Statistical analysis

Data are expressed as mean ± SEM, medians and percentages as appropriate. The Kaplan–Meier method was used to plot the steroid‐free remission time, determined from the time steroids were withdrawn until the first relapse. Patients not having a relapse until the end of the follow‐up period were considered as censored at the time of the last visit. All statistical analysis was performed using prism 6 software. Statistical significance was established at *P* < 0.05.

## Results

### Patient characteristics

The baseline characteristics of all the patients are summarized in Table 1.

**Table 1:** Baseline patient characteristics

	Overall cohort (*n* = 15)
Demographics	
Age at treatment (yr)	27 (18–45)
Male sex	9 (60%)
Caucasian	9 (60%)
Disease characteristics	
<18 years at onset	9 (60%)
Duration of disease (years)	17 (2–27)
Steroid‐dependent/frequently relapsing nephrotic syndrome	8/7
Previous immunosuppression	
Oral steroids	15 (100%)
Calcineurin inhibitors	
Cyclosporine	6 (60%)
Tacrolimus	4 (40%)
Antiproliferative or cytotoxic agents	
Cyclophosphamide	5 (50%)
Mycophenolate mofetil	3 (30%)
Levamisole	3 (20%)
Clinical parameters	
Albumin (mg/dl)	41.4 ± 1.3
Urine protein/creatinine ratio	43 ± 23.7
Creatinine mmol/l	84.4 ± 21.12

Data are presented as median and range or if continuous as mean ± SEM. Categorical data are expressed as numbers and percentages.

Sixty per cent of patients were Caucasian men with disease onset at <18 years of age. The median age of disease onset was 5 years (1–38) and the median age at treatment was 27 years (range 18–46). All patients had previously received long‐term corticosteroid therapy and at least one IS drug such as CNI (*n* = 10), MMF (*n* = 3), levamisole (*n* = 3), cyclophosphamide (*n* = 5). All patients were in remission (mean albumin 41.4 ± 1.3 mg/dl and urine protein/creatinine ratio 43 ± 23.7) and on a CNI at the time of RTX treatment. Twelve patients were on maintenance steroids (mean dose 8 ± 1.85 mg; range 0–20 mg), which were tapered by 3 months following RTX treatment. B‐cell depletion was not achieved in two patients: the first one was not re‐treated and relapsed 4 months later, prior to their second RTX dose, and the second was re‐dosed at 4 weeks.

### Steroid‐free remission and relapses

**Figure 2:**
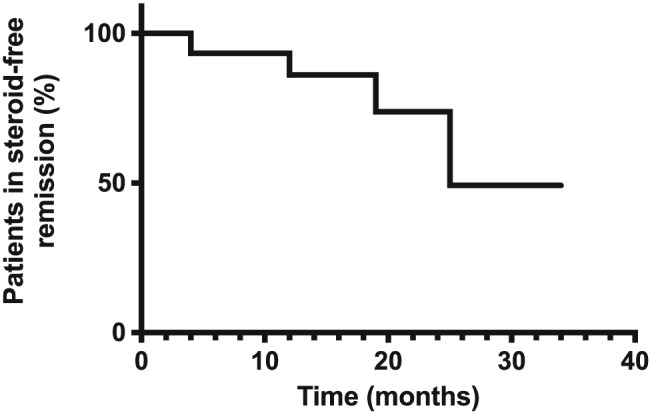
Rituximab achieves prolonged steroid‐free remission. Kaplan–Meier curve showing the percentage of patients in steroid‐free remission after rituximab therapy. Median steroid‐free remission for the group as a whole was 25 months.

Median time of steroid‐free remission was 25 months (range 4–34) for the group as a whole (Fig. 2). Looking at the groups separately based on RTX dosing (Table 2), patients that received two doses of RTX 6 months apart (*n* = 8) remained in steroid‐free remission after a median follow‐up period of 17 months (range 4–31); patients that received one dose of RTX (*n* = 3) remained in steroid‐free remission after a median follow‐up period of 12 months (range 12–16). Patients that received an additional RTX dose at 18 months (*n* = 4) remained in steroid‐free remission for a median follow‐up period of 27 months (range 25–30).

**Table 2:** Follow‐up duration and time to first relapse following rituximab

Patient	Rituximab total exposure (mg)	Follow up (months)	Time to first relapse (steroid free)
1	3000	43	25 months‡
2	3000	37	no relapse
3	3000	33	25 months
4	3000	31	no relapse
5	2000	31	19 months
6	2000	30	no relapse
7	2000	27	no relapse
8	2000	20	no relapse
9	3000†	20	4 months‡
10	2000	17	no relapse
11	2000	17	no relapse
12	3000†	17	no relapse
13	1000	16	no relapse
14	1000	14	12 months
15	1000	12	no relapse

† received additional rituximab after the first dose as did not fully deplete CD19 count.

‡ second relapse 10 months later.

**Figure 3:**
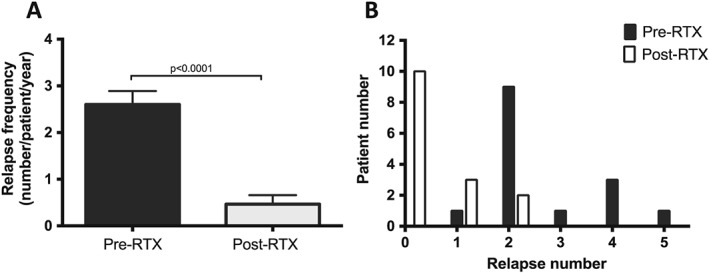
Rituximab (RTX) reduces relapse frequency. (A) Relapse frequency (expressed as mean ± SEM) and distribution (B) in the year before RTX therapy (black bars) and over the entire follow‐up period after treatment (white bars).(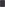) Pre‐RTX. (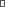) Post‐RTX.

Overall, 39 relapses occurred in this group of patients in the year prior to RTX treatment compared with seven during the entire follow‐up period post‐RTX. Relapse frequency (number per patient per year) decreased from 2.60 ± 0.28, in the year prior to RTX treatment, to 0.46 ± 0.19 (*P* < 0.001) post‐treatment (Fig. 3A,B). One relapse occurred early on in the study period (4 months post‐steroid withdrawal; 6 months post‐RTX treatment) in a patient who did not completely deplete their CD19 count following the first RTX dose. Following the second RTX dose, which achieved CD19 depletion, the patient had a 10‐month steroid‐free remission period before the occurrence of another relapse. Another relapse occurred 19 months post‐steroid withdrawal in a patient who had stopped his CNI (tacrolimus).

### B‐cell counts and concurrent medication

**Figure 4:**
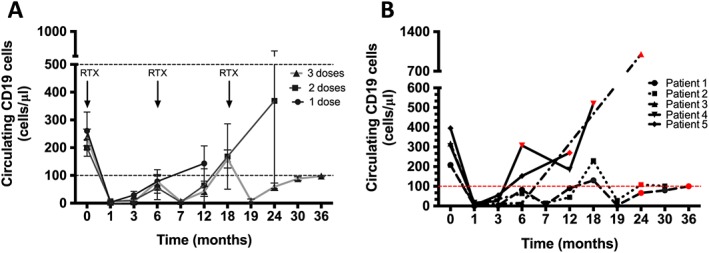
Circulating CD19 B cells following rituximab (RTX) therapy. (A) Circulating CD19 cells remained low (<100 cells/µl) in all patients for at least 6 months after RTX but started to reappear in the circulation 12 months following the last RTX dose. Red lines indicate normal range in our laboratory 100–500 cells/μl. (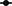) 1 dose. (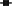) 2 doses. (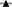) 3 doses. (B) Majority of relapses (red symbols) occurred at a CD19 level greater than 100 cells/ml. (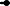) Patient 1. (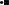) Patient 2. (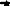) Patient 3. (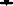) Patient 4. (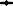) Patient 5.

A single dose of 1 gr RTX successfully depleted CD19 lymphocytes in 86% (*n* = 13/15) of patients. CD19 cells progressively re‐emerged in the circulation 6–12 months following each RTX infusion (Fig. 4A). Most relapses (five out of seven) occurred with CD19 counts of 100 cells/µl or greater (Fig. 4B). Two relapses occurred below this level, in the same patient; the first at a CD19 count of 66 cells/µl and the subsequent one at 99 cells/µl. In the patients that relapsed, average CD19 count at the time of relapse was 351 ± 166 cells/µl.

**Figure 5:**
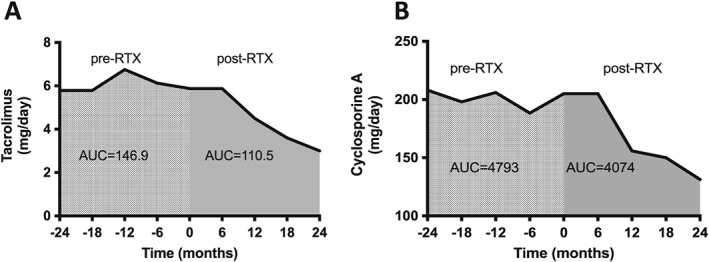
Rituximab (RTX) reduces concurrent immunosuppression. Average daily dose of tacrolimus (A) and cyclosporine A (B) over 24 months pre‐RTX and post‐RTX. AUC, area under the curve.

Steroids were discontinued in all patients within 3 months of the first RTX infusion and were not restarted unless for the treatment of a relapse. Tacrolimus (Fig. 5A) and cyclosporine A (Fig. 5B) average daily dose was compared over a period of 24 months pre‐RTX and post‐RTX. At the 12 month time point, average tacrolimus dose was reduced from 6.75 ± 1.236 to 4.50 ± 1.118 mg/day (*P* = 0.08), and a similar reduction was observed for cyclosporine A (206 ± 20 *vs* 155.83 ± 19.5 mg/day, *P* = 0.08).

### Adverse events

**Figure 6:**
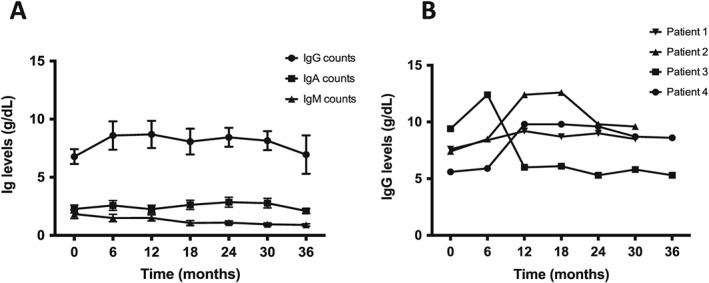
Immunoglobulin levels remain stable following RTX therapy. (A) Immunoglobulin levels were measured at different time points following RTX treatment and did not show significant variation from baseline. (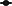) IgG counts. (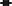) IgA counts. (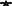) IgM counts. (B) IgG levels in patients receiving three doses RTX remained stable throughout the observation period. (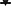) Patient 1. (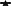) Patient 2. (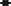) Patient 3. (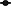) Patient 4.

No serious adverse events were observed during the entire follow‐up period. One episode of lower respiratory tract infection and one episode of gastroenteritis were reported in the 3 months following RTX treatment. Seven patients (46%) experienced Type I hypersensitivity reactions during RTX administration. Repeated RTX administration has been reported to reduce immunoglobulin levels 20, but we did not observe any significant differences in the group overall (Fig. 6A) or in the four patients who received three doses of RTX (Fig. 6B). Average IgG levels at baseline were 6.77 ± 0.637 g/dl and at 1 year 8.725 ± 1.068 g/dl.

## Discussion

Patients with frequently relapsing or steroid‐dependent MCD pose a significant therapeutic challenge. In this prospective, observational study, we describe an effective treatment with RTX that achieves prolonged steroid‐free remission, reduces relapse frequency and consequently exposure to glucocorticoids and CNI.

The rationale for the use of RTX in MCD comes from data suggesting a potential role of B cells in the pathophysiology of NS 21, data showing that RTX may have a beneficial effect on the podocyte cytoskeleton and the recovery of foot process effacement 22, 23 and from studies and case series showing that RTX can induce remission of NS (for systematic review 24). However, as most data on RTX efficacy are derived from retrospective studies, and the observation period in prospective studies to date has been limited to 12 months; data on its long‐term efficacy and safety are lacking. To our knowledge, our study is the only prospective study with an extended observation period of up to 43 months. Our results show that RTX therapy can achieve prolonged steroid‐free remission (median time 25 months) and significantly reduce relapse frequency. The importance of this result is amplified given the young age of the patients and the significant and well‐documented toxicity associated with prolonged steroid and CNI exposure.

Similar long‐term efficacy has been reported in several retrospective series 15, 16, 17, 25. In the study by Guitard *et al*. (*n* = 41 adults) median relapse‐free time post‐RTX was 18 months (range 3–36), although it is not clear if all patients included in the analysis were off steroids. We did not stop concurrent CNI therapy in our population but reduced it by 25% at 12 months and by 25% at 6 month intervals thereafter in eligible patients, which resulted in significant dose reduction. This might have contributed to the prolonged remission period observed as CNIs have been reported to have an anti‐proteinuric effect through stabilization of the podocyte cytoskeleton 26. This strategy was adopted at the time of study initiation as the efficacy of RTX monotherapy in this group of patients was less well studied, and we deemed removal of all IS too high risk in this population. Emerging evidence since then suggests that future trials should address withdrawal of all IS medication following RTX.

As the aim of our study was to extend the period of steroid‐free remission in this group of patients, we used RTX in remission. Whether RTX can be used only as a steroid/IS‐sparing agent or to induce remission without the need for concomitant steroid therapy is an important question that remains unanswered. Earlier studies suggested that relapse rates were lower when RTX was given in remission 16 and that there may be increased urinary loss of RTX in patients with nephrotic range proteinuria 27; the latter observation supported by the finding that RTX‐treated patients with idiopathic membranous nephropathy have lower serum concentrations of RTX compared with patients with rheumatoid arthritis 28. More recent studies however suggest that RTX alone may be sufficient to induce remission in adults 15, 17. Further evidence from, ideally a randomized controlled study, would be needed to clarify this important question.

We elected to use a standard dose of 1 gr for all patients as this dose has been shown to reliably deplete CD19 counts 29 and is substantially more cost‐effective and convenient for patients than multiple dosing. An important observation from our study is that in our cohort, relapses occurred in the main upon reappearance of CD19 counts in the circulation above the lower normal limit of 100 cells/µl with the average CD19 count at relapse being 410 ± 23 cells/µl. Of interest is also the observation that the one patient in our cohort that relapsed early (within 6 months of RTX) had not depleted their CD19 cells fully post‐RTX. This patient also reconstituted their B cells much faster than the rest of the cohort to a level >100 cells/µl and relapsed each time. A similar association between B‐cell recovery and relapse has been reported in other studies. In a Japanese cohort of 25 adult MCD patients, treated with a similar RTX protocol, no relapses were observed during the B‐cell depletion period 19, and in a French cohort of 17 patients, the majority of relapses were observed after the reappearance of CD19 cells 16. Data from the paediatric population also suggest that maintaining prolonged B‐cell depletion with repeated RTX infusions reduces relapse frequency 30, 31. However, relapses are not always associated with B‐cell recovery. In a paediatric cohort of 17 patients, a high proportion remained relapse‐free despite B‐cell recovery 32. This variability might be explained by the heterogeneity of B‐cell subpopulations that reconstitute after RTX. A recent study by Colucci *et al*. found that in a population of 28 paediatric patients, following a single infusion of RTX, transitional B cells, thought to have a more regulatory role 33, appear first in the circulation compared with memory B cells. However, it was the reconstitution of isotype‐switched memory B cells that was a strong predictor of relapse suggesting that monitoring of this B‐cell population might be a useful tool to identify those patients who are at high risk of relapse and who might benefit from re‐treatment 34.

The question of whether long‐term maintenance RTX therapy is beneficial or not has so far not been addressed by previous studies in the adult population. Takei *et al*. showed that patients re‐treated with RTX 6 months after the first infusion, remained in remission for another 6 months 19, the end of the study's observation period. We attempted to address this question by re‐treating patients 12 months after their last RTX infusion (T18), irrespective of CD19 counts. Four patients were treated with this protocol: two relapsed 7 months following the additional RTX dose and the remaining two continued to be in steroid‐free remission until the end of the study period, 31 and 37 months, respectively. Although the patient numbers are small, these results, together with the observation that most relapses occurred upon CD19 cell recovery, suggest that maintenance RTX therapy, keeping B cells below 100 cells/µl, may have a beneficial effect, but further studies comparing different strategies are needed.

An important consideration with repeated RTX infusions is the long‐term safety as well as the development of human anti‐chimeric antibodies directed against RTX. We did not observe any serious infections or persistent hypogammaglobulinaemia in this group. Similar to previous reports, 46% of patients experienced type I hypersensitivity reactions at the time of RTX infusion 24. However, data from the paediatric population suggest that prolonged B‐cell depletion may be complicated by opportunistic infections such as *Pneumocystis jiroveci* pneumonia (PCP), and therefore, PCP prophylaxis might be beneficial 30. Development of anti‐chimeric antibodies has been recently reported in paediatric patients receiving RTX for NS 35. Data from adult patients with rheumatoid arthritis treated with repeated courses of RTX (up to 5) show that 9.2% developed antibodies but did not exhibit decreased efficacy or any additional safety issues 36. Newer, fully humanized B‐cell depleting agents such as ofatumumab and obinutuzumab might be a better long‐term treatment option in this population as they provide prolonged B‐cell depletion with a single dose, and there is less risk of de novo antibody formation.

In summary, our study shows that RTX is an effective method of maintaining a steroid‐free remission in frequently relapsing or steroid‐dependent MCD. The benefit of a safe, long lasting, single agent, in a population of young adults often having problems with non‐compliance and medication burden from the multiple IS agents needed to control the disease and the side effects experienced, make RTX a very attractive treatment alternative to current strategies. Further studies are now required to address specific questions including its role in remission–induction and the benefit of pre‐emptive treatment based on B‐cell reconstitution.
